# HSF1 mediated stress response of heavy metals

**DOI:** 10.1371/journal.pone.0209077

**Published:** 2018-12-19

**Authors:** Christoph Steurer, Noreen Eder, Sarah Kerschbaum, Christina Wegrostek, Stefan Gabriel, Natalia Pardo, Viktoria Ortner, Thomas Czerny, Elisabeth Riegel

**Affiliations:** Department of Applied Life Sciences, University of Applied Sciences, FH Campus Wien, Helmut-Qualtinger-Gasse 2, Vienna, Austria; University of Kansas Medical Center, UNITED STATES

## Abstract

The heat shock response (HSR) pathway is a highly conserved cellular stress response and mediated by its master regulator HSF1. Activation of the pathway results in the expression of chaperone proteins (heat shock proteins; HSP) to maintain protein homeostasis. One of the genes strongest upregulated upon stress is HSPA1A (HSP72). Heavy metals are highly toxic to living organisms and known as environmental contaminants, due to industrialisation. Furthermore, many of them are well-described inducers of the HSR pathway. Here we compare the effect of different heavy metals, concerning their potential to activate HSF1 with a sensitive artificial heat shock reporter cell line, consisting of heat shock elements (HSE). In general the responses of the artificial promoter to heavy metal stress were in good agreement with those of well-established HSF1 target genes, like HSPA1A. Nevertheless, differences were observable when effects of heat and heavy metal stress were compared. Whereas heat stress preferentially activated the HSE promoter, heavy metals more strongly induced the HSPA1A promoter. We therefore analysed the HSPA1A promoter in more detail, by isolating and mutating the HSEs. The results indicate that the importance of the individual binding sites for HSF1 is determined by their sequence similarity to the consensus sequence and their position relative to the transcription start site, but they were not differentially affected by heat or heavy metal stress. In contrast, we found that other parts of the HSPA1A promoter have different impact on the response under different stress conditions. In this work we provide deeper insights into the regulation of HSP72 expression as a well as a method to quantitatively and sensitively evaluate different stressor on their potential to activate HSF1.

## Introduction

Cells need to maintain homeostasis in order to keep up with all their functions. In 1936 Hans Selye [1] described abnormalities and disturbances affecting the cells, which are today known as cell stress. One of the most important pathways activated by cell stress is the heat shock response (HSR) pathway. The HSR is an emergency pathway of the cell and one of the most conserved pathways throughout all living entities. Like the name already suggests, the response can be triggered by elevated temperatures, but not exclusively. Heavy metals, pH changes and many other stress conditions can also induce this response [2][3]. The pathway leads to activation of the heat shock transcription factor 1 (HSF1) and the transient expression of heat shock proteins (HSPs), molecular chaperones that help unfolded or misfolded proteins to regain their native state. The HSP70 protein family is one of the most abundant chaperone class in the cell. It consists of several 70 kDa ATP-binding proteins playing distinct roles as chaperones for newly-synthesized, misfolded or denatured proteins. Furthermore, they prevent the aggregation of unfolded proteins and they promote their re-folding back to the natural state, in addition they also have immunomodulatory functions [4,5]. HSP70 proteins have a variety of chaperone and co-chaperone interaction partners [6] and they also play a role in protein degradation [7]. Several members of the HSP70 family are transcriptional targets of HSF1 and are directly upregulated after cell stress. HSP72 encoded by the HSPA1A gene shows the highest fold-induction after heat stress [8] and is also one of the most upregulated HSPs under a variety of stress conditions [9]. Therefore it is a suitable marker for HSR activity.

HSF1 plays a vital role in the HSR. Upon activation it gets phosphorylated, trimerises and binds to responsive elements in heat shock (HS) responsive genes [10]. These responsive elements are called heat shock elements (HSEs) and consist of 3–6 alternating, inverted pentameric repeats (nGAAn) [11,12]. The HSF1 trimer binds to these repeats and thereby embraces the DNA [13]. Within the HSPA1A promoter there are several HSEs described in the literature [14,15], however, also other pathways and transcription factors (NFκB, NF-Y, Nrf2) were identified to influence HSPA1A transcription [16–18]. This suggests a tightly regulated stress response mediated by this promoter.

Heavy metals, like cadmium, mercury, copper, arsenic and zinc, are known for their toxic potential at already low concentrations. Although some heavy metals are essential trace elements within the human body they have a high potential of biotoxic and ecotoxic effects and are present as pollutants in the air and contaminations in food, soil and water [19,20]. Heavy metals are known to induce the HSR [21], but in addition also activate other pathways, like metallothioneins [2,22]. Although being activated simultaneously, it was shown that these two response pathways do not interfere with each other, when reacting to heavy metals [23]. HSPs have been used previously to monitor heavy metal toxicity [24–26], therefore, the use of a specific HSF1 reporter cell lines could be a valuable tool to analyse cellular reactions to heavy metal exposure.

We previously developed an artificial HSE reporter cell line [27] and demonstrated that a significant higher induction compared to a natural HSPA1A promoter is possible under heat stress conditions [28]. This stable cell line is used in this work to show and compare the activation of the HSR after induction with different heavy metals. Highly sensitive assays for environmental toxins, like heavy metals, can be useful for a wide range of applications, including the prevention of animal testing and toxicology screens for drugs and food additives. Furthermore, we also investigated the roles of the individual HSEs within the HSPA1A promoter under different stress conditions to get deeper insights into the regulation of HSP expression.

## Material and methods

### Plasmids

For transient transfection experiments pMlucF 6HSE [28] was adapted by changing the Fos minimal promoter to an artificial minimal promoter, containing a TATA box and an initiator element (tataaaattctcattcagccgataccgtctcactct). Furthermore, firefly luciferase was changed to NanoLuc [29] with protein destabilizing sequences (NlucP; derived from pNL1.2; Promega) and RNA destabilizing sequences (NlucPAU, containing two tttatttatttatttattta elements within the 3’ UTR) [30] to create pMNluc PAUM. The different HSEs were inserted into the multiple cloning site upstream of the minimal promoter (see S1 Table). pMNluc PAUM HSPA1A was created by changing firefly luciferase to NlucPAUM in the pMluc HSP72 promA [28]. The mutations in the HSPA1A promoter were generated with PCR (HSE1m, c**T**aaaccc**A**tg**T**aatat**C**cc; HSE2m, a**T**aagact**A**tg**T**agagtt**A**t; HSE3m, g**T**aacttt**A**ca**T**tacttt**A**c).

### Cell culture, transfection and statistical analysis

HEK293 T-Rex cells were purchased from Invitrogen. C5 cells contain a bidirectional artificial heat shock promoter with 8 times mulitmerized HSEs driving the expression of firefly luciferase and GFP and were previously described [27]. XshHSF1-5-13 HSF1 knock-down cells were generated by lentiviral transduction of HEK293 T-Rex cells with MISSION shRNA lentiviral particles (TRCN0000318712, Sigma Aldrich) and single clone selection. All cell lines were kept in an incubator at 37°C with 5% CO_2_ in Dulbecco’s modified eagle medium (DMEM) with 4.5 g/L glucose, Na-Pyruvate L-glutamic acid, 10% fetal bovine serum and 1 x Penicillin / Streptomycin. DMEM containing all the before mentioned substances will be called DMEM complete hereafter. For experiments in 96-well plates, the plates were coated with polyethyleneimine (PEI) [31] before seeding. Transient transfections were conducted as described in [28]. For microscopy cells were seeded in a 6-well plate and treated with cadmium in the same way as for luciferase reporter assays. After 48 h cells were stained with PI (1 ng/μL) for 30 min before imaging with a Zeiss Observer microscope. P-values were calculated by the Student’s t-test. Statistical significance: *p ≤ 0.05; **p ≤ 0.01; ***p ≤ 0.001.

### Luciferase and viability measurement

The experiments were performed as previously described [32], except for single luciferase measurements, where the firefly substrate or the NanoLuc substrate (6.25 mM Tris pH 7.5 and 3 μM coelenterazine) were injected, followed by immediate measurement and injection of stop solution.

### Cell treatment

NaCl (Carl Roth), ZnCl_2_ (Carl Roth), AgNO_3_ (Carl Roth), HgCl_2_ (Carl Roth), NiCl_2_ (Applichem), AsNaO_2_ (Sigma Aldrich), NiCl_2_ x 6 H_2_O (Sigma Aldrich), CdSO_4_ x 8/3 H_2_O (Sigma-Aldrich), CuSO_4_ (Sigma Aldrich), Pb(NO_3_)_2_ (Carl Roth) and guanidine hydrochloride (Carl Roth), respectively D-Sorbitol (Carl Roth) were dissolved to a concentration of 1 M, stored at—20°C and diluted in DMEM complete. For continuous treatment, the medium in the well was removed and replaced with inducer, diluted in DMEM complete for 6 h or 24 h. For 1 h induction cells were induced for 1 h with DMEM complete containing heavy metals. After 1 h this medium was removed and replaced fresh DMEM complete.

### Western blot

Whole cell protein extracts and Western blots were performed as described in [33], primary antibodies anti-HSF1 (51034-1-AP, Proteintech; dilution 1:1000) and anti-GAPDH (sc-25778, Santa Cruz; dilution 1:5000) and secondary antibody HRP-conjugated anti-rabbit antibody (sc-2357, Santa Cruz; dilution 1:5000).

### Flow cytometry

Flow cytometry was performed with a Beckman-Coulter Cytoflex. Cells were seeded in a 6-well plate. After induction and recovery, cells were trypsinized, washed once with PBS, resuspended in 500 μL PBS and then analysed directly for GFP signals. For apoptosis assay, cells were resuspended in 100 μL Annexin binding buffer (140 mM NaCl, 2.5 mM CaCl2, 10 mM Hepes pH 7.5) with 3 μL Annexin V-FITC (0.1 μg/μL stock) and 1 μL PI (1 μg/μL stock), or 1 μL 7-aminoactinomycin D (7-AAD, 1 ng/μL stock) was applied for 15 minutes after washing with PBS. Before apoptosis analysis, 400 μL Annexin binding buffer was added.

### qPCR

TaqMan qPCR for HSPA1A/B, GAPDH and luciferase was previously described in [28]. SYBR green qPCR was performed as described in [33], with following primers DNAJB1 (NM_001300914.1) fwd: CCTCCAACAACATTCCAGCTGA rev: ACGTTCACTGTGCAGCCACAC HSPB1 (NM_001540.4) fwd: GCGTGTCCCTGGATGTCAACCACTT rev: ACTTGGCGGCAGTCTCATCGGA HSPA6 (NM_002155.4) fwd: ACGTGCTCATTTTTGACCTGGGTGG rev: AAGCGGGCACGAGTGATGGA

### Trypan blue

2 x 10^5^ C5 cells per well were seeded and then incubated for 3 days at 37°C. Cells were then treated with different concentrations of heavy metals. For negative control cells were incubated without addition of the reagent. 24 h after treatment the death rate was determined with 0.2% trypan blue. Cells were counted with a Cellometer (Nexcelom Bioscience, Auto T4). At least 1000 cells were counted per sample. Viability was determined by counting dead cells (blue cells) compared to the total cell count for each sample. Curve fitting was done using the least-squares method (described in [32]).

## Results

### Heavy metal induced HSR analysed with a HSE reporter cell line

**Fig 1:**
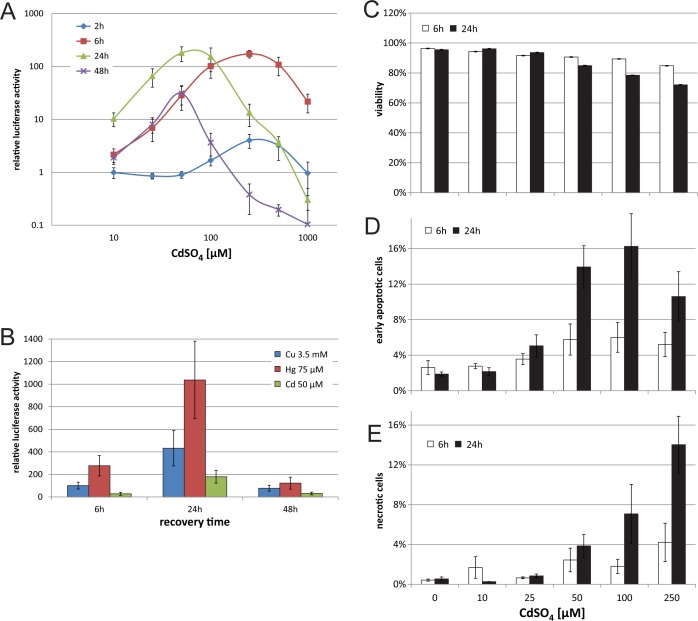
Heavy metal induced HSR in C5 cells. (A) C5 cells were treated with the indicated concentrations of CdSO_4_ in DMEM complete for 1 h. Then the cells were washed once and recovered in DMEM complete for different time points (2, 6, 24, 48 h). Y-axis shows relative luciferase activity compared to untreated control cells. (B) C5 cells were treated with CdSO_4_ (Cd, 50 μM), CuSO_4_ (Cu, 3.5 mM) or HgCl_2_ (Hg, 75 μM) in DMEM complete for 1 h. Then the cells were washed once and recovered in DMEM complete for 6, 24 or 48 h. Y-axis shows relative luciferase activity compared to untreated control cells. (C-E) For viability assay with flow cytometry, HEK293 cells were treated with different concentrations of CdSO_4_ in DMEM complete for 1 h. Samples were taken after 6 h and 24 h, then stained with AnnexinV-FITC and 7-AAD. Y-Axis shows percentage of viable cells (no 7-AAD staining) compared to total cell count (C), early apoptotic cells, positive for AnnexinV-FITC (D) and of necrotic cells, positive for 7-AAD (E). All values show means of at least three independent experiments, luciferase measurements were performed with 6 technical replicates each. Error bars indicate SEM.

In previous work [28] we characterized the HEK293 C5 cell line, containing an artificial HSE-driven reporter, with classic HS experiments. We could demonstrate, that this minimal artificial HS reporter can reflect the HSR, similar to the endogenous target gene HSPA1A, but with higher induction rates. In this work we were interested if this is also true for other well characterized inducers of the HSR, like heavy metals. To evaluate the kinetics of our HSE reporter cell line under heavy metal stress, we first chose the well-known inducer CdSO_4_. In agreement with previous experimental settings, based on heat treatment, we incubated the cells with heavy metals for a short time (1 h) and then analysed the HSR after different recovery times. To cover various cellular reactions, we selected a wide range of CdSO_4_ concentrations (from 10 μM to 1 mM) and analysed the response at early (2 h and 6 h) and late time points (24 h and 48 h) in Fig 1A. The results showed induction for all tested time points. The maximum induction ranged from 4-fold (2 h, 250 μM) to ~180-fold (6 h, 250 μM and 24 h, 50 μM). The earlier time points showed a peak of luciferase activity at comparably higher CdSO_4_ concentrations, at later time points and high concentrations the luciferase signal decreased below the un-induced control level. Except for the 2 h measurement, all time points showed good induction levels at concentrations of 25 μM (from 7 to 65-fold). In general, the highest activities at the lowest concentrations were measured after 24h. This peak of activation, after 24 h recovery, was also seen for the heavy metals copper (Cu) and mercury (Hg) (Fig 1B), both well-known inducers of the HSR, and correlates with Hsp72 protein level (S1 Fig). Although the quantity of the reporter activation differed between the heavy metals, the kinetics were the same with a slight activation after 6 h, peak activation after 24 h, followed by a decrease after 48 h.

To correlate the reporter signal to the general viability of the cells, we performed viability assays with flow cytometry. When looking at general viability (cells with intact cell membrane, Fig 1C), the viability decreased with time and increasing concentrations of CdSO_4_. After 6 h recovery, the cells were hardly affected (viability with 250 μM CdSO_4_ at 85%), but after 24 h recovery, the viability steadily dropped, starting from 50 μM CdSO_4_. With a concentration of 250 μM, the viability was at 72% after 24 h. For both time points the drop of viability below 75% marks the point where also the reporter luciferase signal started to decrease (Fig 1A). This indicates that the kinetics of the reporter at high concentrations is mainly influenced by the loss of viable cells. To look further into detail of the cell fate after a 1 h heavy metal insult, we also analysed early apoptosis (Fig 1D) and necrosis (Fig 1E). The results show that cells first started to become early apoptotic at 50 μM (6% after 6 h, 14% after 24 h) and then had a peak at 100 μM (16% after 24 h). With a further increase of CdSO_4_ concentrations, the number of early apoptotic cells decreased, whereas cells also started to become more necrotic (14% after 24 h, 250 μM CdSO_4_). Taken together, the reporter shows induction at very low concentrations, where the cells are still highly viable, indicating that the HSR reporter is activated specifically, rather than being induced due to general cell stress.

**Fig 2:**
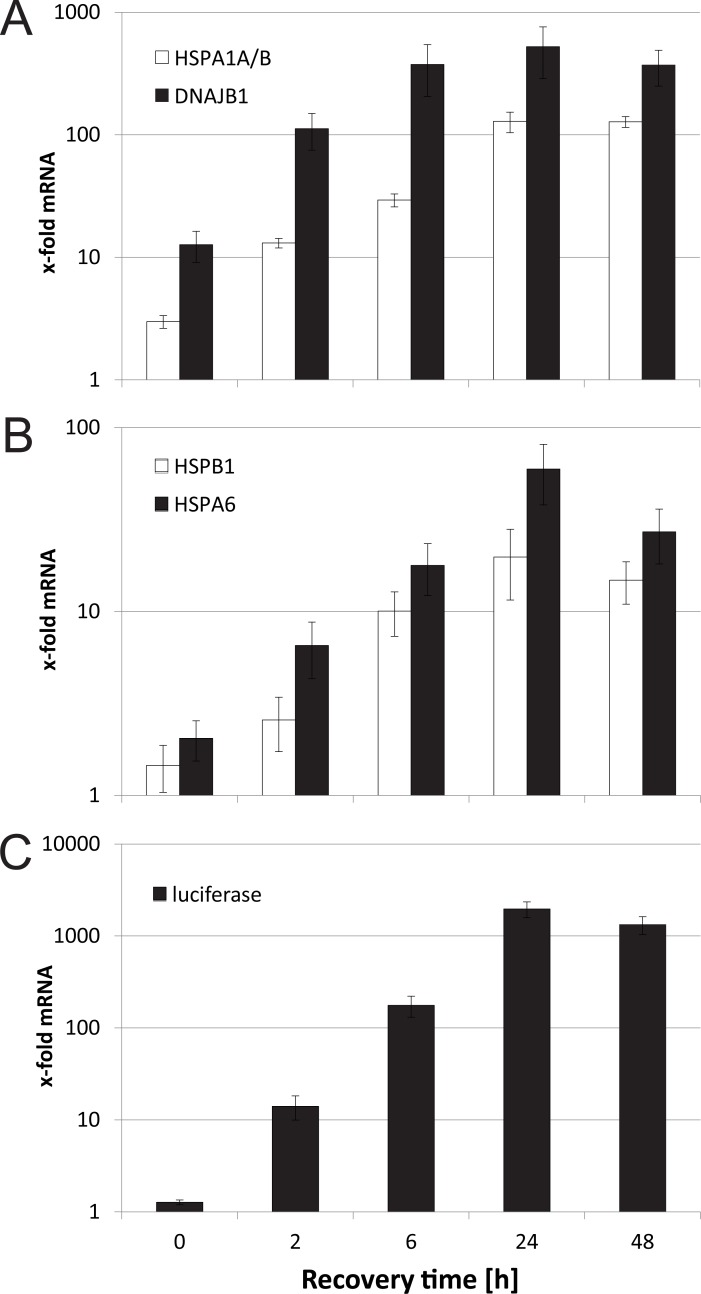
HSF1 target gene expression on mRNA level. C5 cells were treated with 250 μM CdSO_4_ for 1 h, then washed once, and afterwards recovered for different time periods (2 h, 6 h, 24 h and 48 h), before mRNA harvest. Quantitative PCR was performed for HSPA1A/1B and DNAJB1 (A), HSPB1 and HSPA6 (B) and firefly luciferase (C). GAPDH was used for normalization. Y-axis shows x-fold mRNA levels compared to untreated control cells. All values show means of at least three independent experiments. Error bars indicate SEM.

After proving the general functionality of the reporter under heavy metal stress, we asked how well the artificial reporter represents the endogenous HSR. Therefore, we compared mRNA levels of the HSE-driven reporter (luciferase) with endogenous targets of HSF1 (HSPA1A/B, DNAJB1, HSPB1 and HSPA6) at different time points. We selected 250 μM CdSO_4_ to see activation at all time points. The two targets in Fig 2A, HSPA1A/B (HSP72) and DNAJB1 (HSP40), showed similar kinetics upon CdSO_4_ treatment, but differed in their absolute values. DNAJB1 was strongly induced, starting directly after the CdSO_4_ treatment (13-fold) and high values after 6 h and 48 h (375- and 370-fold, respectively). Nevertheless, after 24 h the induction was at its peak (525-fold induction). HSPA1A/B mRNA levels were lower, but also showed peak activity at 24 h (129-fold). Two other targets (Fig 2B), HSPB1 (HSP27) and HSPA6 (HSP70B’), showed lower induction rates in general, but with the same dynamics, peaking at 24 h (20-fold and 59-fold respectively). Both genes showed weak induction at the 0 h time point.

When the luciferase protein levels (Fig 1A) are compared to the luciferase mRNA levels (Fig 2C), a much faster decline is observed at 24 and 48 h. This is due to the internal normalization of the mRNA levels to the housekeeping gene GAPDH, whereas massive cell death reduces the non-normalized protein levels. At early time points of 2 h and 6 h, where viability is hardly affected, the induction levels of protein and mRNA are very similar (4-fold compared to 14- fold for 2 h and 173-fold compared to 176-fold for 6 h, respectively). With nearly 2000-fold induction after 24 h recovery, luciferase mRNA shows an almost 4 times higher induction level compared to the best target gene DNAJB1 (525-fold induction). In general, however, the luciferase measurements of the artificial promoter well recapitulate the kinetics of the HSR on endogenous target genes.

Taken together, the artificial reporter, integrated into the C5 cells, reflects the endogenous HSR after heavy metal stress, with the highest inductions after 24 h. In contrast to the endogenous target genes, the main advantage of the artificial promoter is, that it solely reacts to the activity of HSF1 making it possible to restrict the analysis to a single pathway. This could also be demonstrated by performing a CdSO_4_ experiment in an HSF1 knock-down cell line, where no induction could be seen for the HSE reporter after treatment (S2 Fig). Furthermore, the artificial construct shows higher induction levels than the other tested target promoters.

### Role of individual HSEs in the HSPA1A promoter

**Fig 3:**
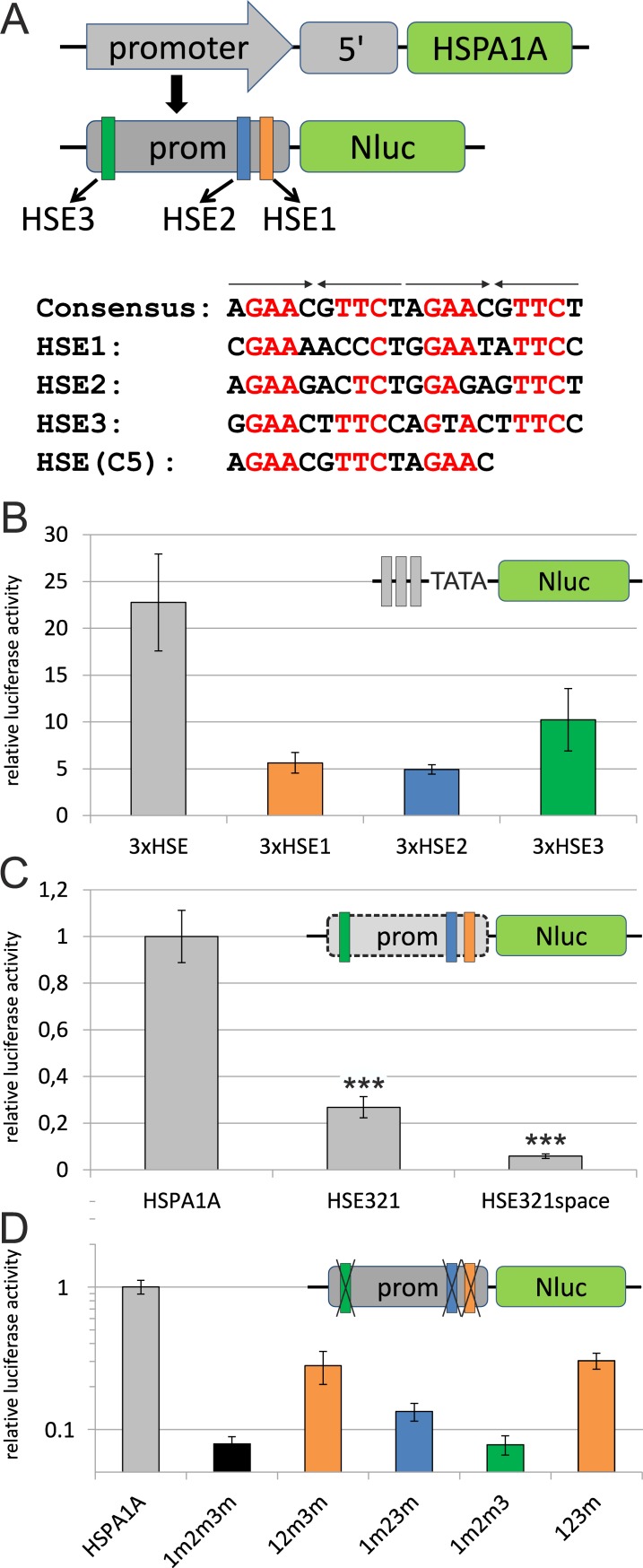
Mutations of HSEs in the HSPA1A promoter. (A) Scheme of the luciferase reporter construct, derived from the natural HSPA1A promoter, with NanoLuc (Nluc) inserted downstream of the transcription start site. The three specific HSF1 binding sites (HSEs) are marked in different colours. Alignment shows HSEs compared to the consensus sequence, the pentameric elements are indicated by arrows, matches with the core sequences [35] of the consensus are highlighted in red. The HSE used in C5 cells is derived from the consensus HSE. (B-D) Effects of individual HSEs on HSPA1A promoter activity analysed by transient transfection experiments in HEK 293 cells; induction with 50 μM CdSO4 for 1 h, followed by 6 h recovery. All constructs are in the same plasmid background (pM Nluc PAUM; see S1 Table); (B) Isolated HSEs 3-fold multimerised; (C) combinations of the HSEs with or without spacers, compared to wildtype HSPA1A and (D) mutations of HSEs in the context of the promoter, (m) following the number of the HSE in the name indicates a mutation. P-values in (C) were calculated relative to HSPA1A. Values shown are means of at least 3 independent experiments with 6–12 replicates each. Y-axis shows relative luciferase activity compared to untreated control cells (B-D) in relation to HSPA1A (C,D). Error bars indicate SEM.

The good correlation between the artificial HSE promoter and the endogenous HSF1 target gene expression indicates that the HSEs of the HSP promoters are the most essential elements for the activation with heavy metals. We therefore selected the promoter of HSPA1A to study the roles of the individual HSEs within the promoter in detail with mutational analysis. HSPA1A was chosen as a prototype HSP because it was already demonstrated to be activated by a variety of heavy metals [23] and further is known to be among the best induced HSP after stress insults [8,9]. Searching through the promoter sequence we identified three motifs (here called HSE1, HSE2 and HSE3) showing high similarity with the HSE consensus sequence [12]. All three motifs have been described in the literature [14,15,34]. They show considerable deviations in their sequence, however, best matches are seen within the core sequences of the pentameric repeats (Fig 3A). The highest overlap with the consensus sequence is seen for HSE3. In addition, their distance to the transcription start site (TSS) might be considered for their role in HSR activation [32]. HSE1 is the nearest (-61 bp from TSS), HSE2 is close to HSE1 (-154 bp) and HSE3 is the one furthest away (-687 bp). For the analysis these elements were either tested isolated, in a trimerised version to mimic their number within the natural promoter, or they were mutated within the HSPA1A promoter context. For detection, these sequences were inserted into a NanoLuc reporter plasmid, upstream of a TATA box. The mutations were introduced into the core-sequences of all pentameric HSE repeats (described in the Materials and methods section).

In order to investigate the role of the different HSEs the cells were induced with 50 μM CdSO_4_ and analysed after 6 h recovery time, as these conditions resulted in strong reporter activation without compromising the viability to a large extent (Fig 1). The multimerised binding sites were compared to the HSE consensus sequence (3xHSE), also present in the C5 cell line (Fig 3B). As expected, the optimised HSE reporter shows the strongest effect in this context (22.8-fold). HSE3, which is the most distant binding site, shows the second strongest induction after cadmium treatment (10.2-fold), followed by HSE1 and HSE2 (5.6-fold and 4.9-fold induction, respectively). This result is in good agreement with the match of the HSEs to the consensus sequence, which is highest for HSE3.

The isolated HSEs therefore strongly react to the CdSO_4_ treatment, indicating that this heavy metal stress response is mediated by HSF1. However, sequences within the HSPA1A promoter could modulate their response. We therefore directly compared the HSPA1A promoter construct with a combination of all three individual HSEs (HSE321). And indeed, when omitting the sequence between the HSEs in the endogenous context, the reporter activity is reduced to one third compared to the wild type HSPA1A promoter (Fig 3C). To exclude simple sterical hindrance by putting the 3 HSEs right next to each other, we further created an artificial promoter replacing the natural sequences between the HSEs with artificial non-reactive sequences (HSE321space), corresponding to their length in the natural promoter. The result was even weaker reporter activation, indicating that the extended space between the single HSEs and the TSS further reduces the ability of the promoter to react to heavy metal stress.

As a complementary approach we analysed the HSEs within the context of the natural promoter by mutating individual binding sites (Fig 3D). Here again all mutations were compared to the natural HSPA1A promoter containing wild type HSEs. When all HSEs were mutated (1m2m3m), only 8% activity was left. Interestingly, no difference was detected when HSE1 and HSE2 were mutated and only HSE3 (1m2m3) was left as binding site. Slightly higher induction levels (13%) were detected when only HSE2 was not mutated (1m23m). The strongest activity for a single functional binding site was observed for HSE1 (12m3m; 28%). Addition of a second functional HSE (HSE2; 123m), only slightly increased the activity to 30%. This pattern of the mutated HSEs in the natural promoter context could also be observed with other heavy metal treatments (Hg, Cu) or heat treatment (S3 Fig), indicating that all three heavy metals and heat stress activate the different HSEs in a comparable manner. If the isolated HSEs are compared to the point mutations in the natural context of the promoter, a different picture appeared. Isolated HSE3 showed the strongest induction, but when only HSE3 was left as functional binding site in the natural promoter, no activity gain was detected compared to the mutated version. HSE1 on the other hand, showed the opposite behaviour. If isolated it showed only weak induction, but within the natural promoter it resulted in the strongest induction of all 3 HSEs. HSE2 seems to be right in the middle between the other two HSEs, without a significant change of induction, whether it is isolated, or the other two are mutated in the natural promoter. Apart from the HSEs, the rest of the promoter seemed to play a synergistic role for the induction of HSPA1A transcription. To check if the observed pattern for the single HSEs is unique to heavy metal induced HSR, we also performed the experiments with HS (S4 Fig). Beside a higher induction throughout all experiments, heat treatment resulted in a marked difference for the activity of HSE321 compared to the natural promoter. Whereas the compressed version, lacking all promoter sequences between the HSEs, was strongly active after HS, little induction was observed after CdSO_4_ treatment (compare Fig 3C and S4B Fig). The opposite behaviour was observed for the natural promoter. This indicates that the parts of the promoter outside of the HSEs play only minor or even repressive roles during heat induced HSR.

### C5 cells tested with different heavy metals

**Fig 4:**
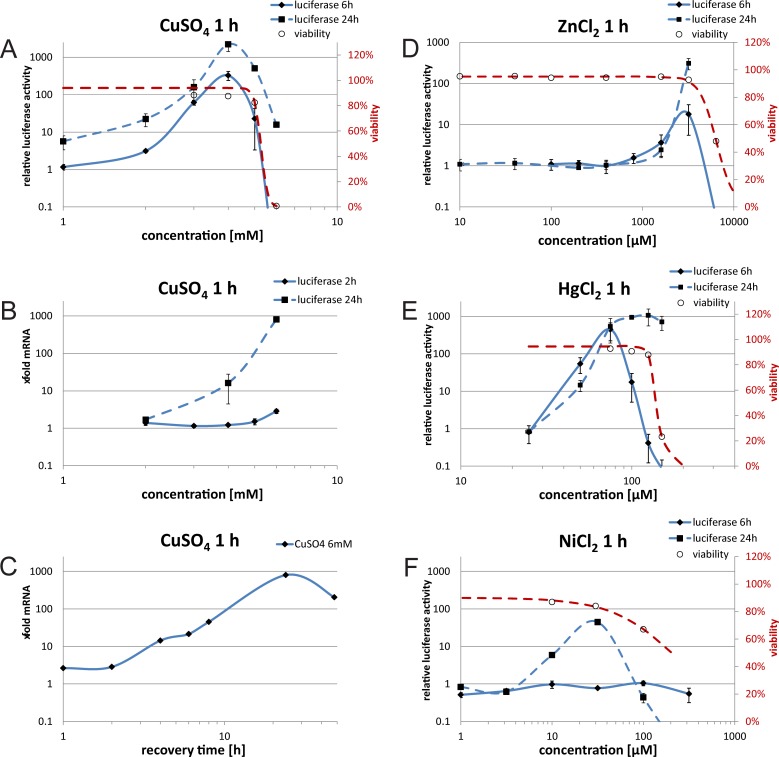
Analysis of C5 cells with different heavy metals. (A-C) C5 cells were treated with CuSO_4_ for 1 h. Then the cells were recovered for up to 24 h. (A) Luciferase reporter assay, Y-axis shows relative luciferase activity compared to untreated control cells for 6 h and 24 h. Viability (curve fitted red dotted line, right y-axis) was measured with trypan blue. (B,C) qPCR, Y-axis shows x-fold luciferase mRNA compared to untreated control cells relative to GADPH mRNA. (B) mRNA induction after 2 and 24 h; (C) time course of mRNA induction for 6 mM CuSO_4_ treatment. (D–F) Luciferase reporter assay, C5 cells were treated with different inducers for 1 h and 6 or 24 h recovery. Y-axis shows relative luciferase activity compared to untreated control cells. X-axis shows concentration of heavy metals. Viability (curve fitted, red dotted line, right y-axis) was measured with trypan blue. (D) ZnCl_2_ (E) HgCl_2_ (F) NiCl_2_. All values show means of at least three independent experiments. Error bars indicate SEM.

After characterising the response of the C5 cell line to CdSO_4_, as our model substance for heavy metals, we expanded our study to other heavy metals (Fig 4 and S5 Fig). We again looked at early reactions to a 1 h heavy metal treatment (6 h recovery for luciferase measurements and 2 h for mRNA) and late reactions (24 h for luciferase and mRNA). Similar to CdSO_4_ (Fig 1A), the 24 h recovery time led to higher maximum induction (Imax), compared to 6 h. The viability at the peak levels, for all tested heavy metals, was more than 80%, but dropped dramatically at higher concentrations. The shape of the curves is similar between CdSO_4_ (Fig 1A, Imax 24 h 173-fold at 50 μM), CuSO_4_ (Fig 4A, Imax 24 h 2222-fold at 4 mM) and HgCl_2_ (Fig 4E, Imax 24 h 1064-fold at 125 μM), although CuSO_4_ needs concentrations almost 100-fold higher than CdSO_4_. Also the range of concentrations resulting in an activation of the HSR differs between the heavy metals. Whereas for CdSO_4_ activation can be detected between 10 and 1000 μM (Fig 1A), it is only detectable between 1 and 6 mM for CuSO_4_ (Fig 4A) and between 50 and 120 μM for HgCl_2_ (Fig 4E). For ZnCl_2_ (Fig 4D) there is only a narrow peak seen at a concentration of 3200 μM (18-fold relative luciferase activity after 6 h, 310-fold after 24 h). Furthermore, cells induced with NiCl_2_ showed a different behaviour, with no induction after 6 h. Only after 24 h there is a 45-fold relative luciferase activity with 31.6 μM of NiCl_2_. For CuSO_4_ we additionally analysed mRNA levels of the luciferase reporter gene. When comparing the luciferase protein level (Fig 4A) and the mRNA level (Fig 4B) at different CuSO_4_ concentrations it became evident, that luciferase protein measurements were more sensitive than mRNA quantification (22-fold activation of protein compared to1.7-fold activation of mRNA for 2 mM CuSO_4_ after 24 hrs). When looking at time course experiments with 6 mM CuSO_4_ (Fig 4C) the kinetics is quite similar to CdSO_4_ (Fig 2A), with a steady increase in activation up to 24 h (802-fold). Concordant, after induction with other heavy metals (Hg, Zn, Ni) the mRNA of HSPA1A showed peak activation at similar concentrations as seen in the luciferase assay but with higher sensitivity (S6 Fig). Taken together, the results for the different heavy metals show that even with a short treatment of 1 h, the cells continuously increase their HSR activation up to 24 h.

### Sensitive detection of heavy metals with HSE reporter constructs

**Fig 5:**
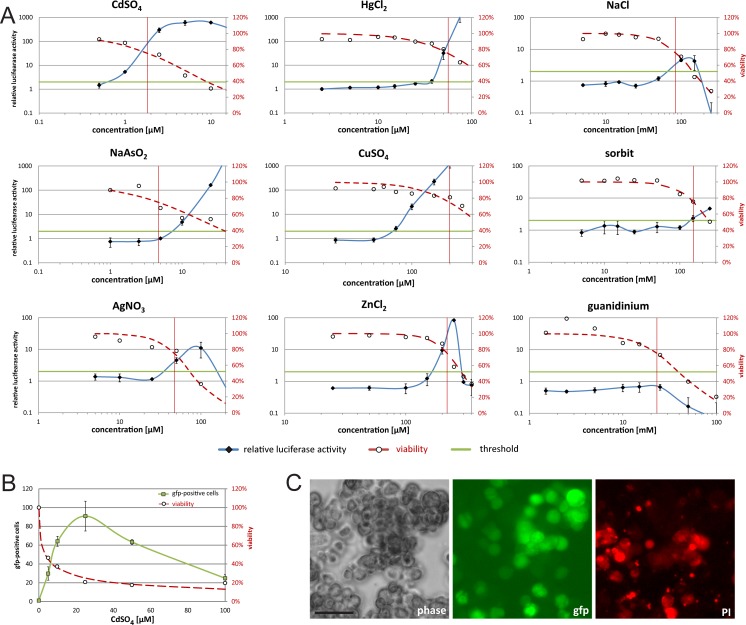
Detection of heavy metals with C5 cells. (A) C5 cells were incubated with different potential inducers for 24 h. All substances were diluted in complete medium. Viability (curve fitted, red dotted line, right y-axis) was measured with resazurin assay (A) or PI staining (B) and is shown relative to untreated cells. Red lines delimit regions above 75% viability. Left y-axis shows relative luciferase activity compared to untreated control cells. Green line (at relative luciferase activity of 2) indicates a threshold for significant induction. All values show means of at least three independent experiments with 12 technical replicates per plate. Error bars indicate SEM. (C) Pictures of C5 cells taken with fluorescence microscopy (scale bar 50 μm) after treatment with 25 μM CdSO_4_ for 48 h. From left to right: phase contrast, GFP, PI staining. Under the same conditions as described in (A), percentage of GFP positive cells and dead cells (PI positive) was determined with flow cytometry (B).

Next, we wanted to know how sensitive the C5 reporter cell line is for the detection of low amounts of heavy metals. Based on the result that the HSF1 activity steadily increased up to 24 h, we switched to a 24 h continuous incubation. In order to determine the lowest effective concentration (LEC) of the heavy metals, we set the threshold for the relative luciferase activity to 2 (based on a 3-fold SD distance from 1). In parallel to the luciferase measurements, a viability assay was performed with resazurin. This was also considered for the evaluation. As the HSR also reacts to unspecific stress factors at highly toxic concentrations, a threshold of 75% was used for the viability. We tested 6 different heavy metals (Cd, As, Ag, Hg, Cu, and Zn) and 3 non-heavy metal controls (NaCl, sorbitol and guanidinium) (Fig 5). All of the tested heavy metals induced the luciferase reporter significantly. The controls NaCl and sorbitol showed an induction of the reporter at high concentrations (LEC of 100 mM and 150 mM respectively), where the viability for sorbitol is already below 75%. Guanidinum shows no effect on the reporter activation up to concentrations where the cells die. The heavy metals strongly differed in their induction levels. CdSO_4_ passes the threshold already at 1 μM. HgCl_2_ is slightly above the threshold at a concentration of 37.5 μM. AsNaO_2_ induces the C5 cells significantly at a concentration of 10 μM (4.8-fold induction), but the viability is only at 56%. This is the only heavy metal where the viability threshold of 75% at the LEC is undershot. CuSO_4_ (75 μM; 2.6- fold), AgNO_3_ (50 μM; 4.5-fold induction) and ZnCl_2_ (200 μM; 9.5- fold) all show a LEC, before the viability drops below 75%, which leaves the assay with the C5 cells as a reliable indicator for a HSR, considering that the non-heavy metal inducers have their LECs around 100 mM. We performed the comparison of the LECs for the different heavy metals with a luciferase assay, as it allows a sensitive, quantitative comparison between the substances. However, as the C5 cells contain a bidirectional HSE promoter, also expression of GFP upon induction [36] can be used for quantification. We therefore performed flow cytometry analysis (Fig 5B) and fluorescence microscopy (Fig 5C), to detect GFP positive cells. In both assays we surveyed cell viability by looking at PI positive cells. The results of the flow cytometry support well the data from the luciferase measurement. A steady increase in the number of GFP expressing cells is seen until at 25 μM CdSO_4_ (viability ~20%) almost 100% of the cells show GFP signals. The decrease after 25 μM suggests that at very high CdSO_4_ concentrations cells die, without turning on the HSR. The fluorescence microscopy picture at 25 μM also shows high GFP expression within the cells. The bidirectional HSE promoter integrated into the C5 cell line therefore allows not only analysis via luciferase measurement, but also with flow cytometry, microscopy or a fluorescent plate reader.

## Discussion

HSF1 is the key player of the HSR pathway. Target genes like HSPA1A are strongly induced by this transcription factor, however, their promoters typically integrate signals from several different signalling pathways [17,18]. HSPA1A reporter cell lines have already been used to detect cellular stress [37–39] and the response to heavy metals [40], however, in this study we intended to concentrate on the effect of heavy metals solely on the HSR. An ideal tool for this purpose is the artificial HSE promoter, consisting exclusively of consensus HSEs [28]. A comparison of the activity of this artificial promoter, to that of HSP target genes, indicates a largely parallel regulation after heavy metal treatment, peak inductions however, were strongest for the artificial version (Fig 2). This is most likely due to the high multimerisation of HSEs, not appearing in natural promoters.

When we analysed the kinetics, CdSO_4_ treatment, as well as that of other heavy metals, resulted in a steady increase of HSR activity, with peak levels around 24 h (Figs 1B, 2 and 4C). Despite a short incubation time of 1 h, the heavy metal treatment caused increasing HSF1 induction over many hours. These kinetics are in sharp contrast to heat treatment, which typically results in early responses within a few hours [28]. At high temperatures a delay in the response is observed, probably due to a general block of translation after severe heat stress [41]. In contrast, the heavy metal induced HSR continuously increased. Heavy metals and heat are thought to denature cellular proteins and thus activate the HSR. Whereas heat acts rather transiently, heavy metals seem to continue the denaturing action on the cells over long periods. Alternatively, the release of the heavy metals from the cells might be a slow process.

A surprising difference was observed for the activity of the HSPA1A and the HSE promoters to different stress conditions. While the artificial promoter strongly reacted to heat (S4 Fig), the HSPA1A promoter showed comparatively higher induction rates for heavy metals (Fig 3). Comparisons of these stress conditions so far, concentrated on the differential expression of HSP target genes [21][42][24]. Lack of HSF1 strongly diminishes the response of target genes [43] (S2 Fig), thus underscoring the prime dependence of these promoters on HSF1. However, the potential contribution of other stress pathways is less clear. The HSPA1A promoter integrates a number of signalling pathways [17], including a potential metallothionein binding site [14]. In addition, heavy metals can mediate their effects indirectly by inducing other pathways, for example via oxidative stress [44]. The observed differences in activity for heat and heavy metal stress suggest that the HSPA1A promoter contains elements reacting specifically to heavy metals, whereas a reduction to its HSEs (HSE321) strongly diminishes this activity (Fig 3B). On the contrary, heat stress preferentially seems to react on the HSEs (S4 Fig).

Another question is if HSEs can react differentially to various stress conditions, or whether their activity primarily relies on their affinity for HSF1, together with their position and context within the promoter. We therefore analysed the contribution of individual HSEs in the HSPA1A promoter by performing a detailed analysis of the HSPA1A promoter (Fig 3, S3 and S4 Figs). When we analysed isolated HSEs, we found that all three elements where bound by activated HSF1 after cadmium or heat treatment. HSE3, which is closest to the consensus sequence [12], showed the highest induction, indicating a high affinity for HSF1. In the natural context, the ablation of this binding site had the weakest effect. This can be explained by its long distance from the transcription start site [32]. We could not see any differences between the single HSEs in respect to the different HSR inducers [34], indicating that the affinity of HSF1 is strictly depending on the sequence similarity of the HSEs to the consensus sequence and does not depend on the mode of HSR activation. Applying various stress factors to the HSE mutants a uniform pattern of activity was observed (S3 Fig), indicating that heat as well as different heavy metal stress conditions target the different HSEs in the same manner. A complete depletion of all three HSEs, in the context of the promoter, inhibited reporter activation entirely for both, heat and heavy metal treatment. The results indicate, that for heat stress HSF1 is, by far, the most important transcription factor, whereas for heavy metal stress the role of other transcription factors binding to the HSPA1A promoter [17,18,45] contribute important synergistic effects.

In order to explore the potential use of the HSE reporter cell line for the sensitive detection of heavy metals, we switched to a 24 h exposure. To prevent non-controlled conditions we included a viability assay and limited the analysis to a 75% survival rate of the cells. Using these conditions, Cd-, Hg-, Cu-, Ag-, and Zn-ions were reliably detected (induction ≥ 2-fold), whereas NaAsO2 resulted in high reporter activation only at toxic levels. This is remarkable for arsenic which is known to be a very potent HSR inducer [24], and also gave the highest maximum induction in our assay. Nevertheless, general unspecific cell stress alone (demonstrated in the case of guanidinum) does not activate the HSE reporter. The non-heavy metal controls NaCl and sorbitol showed reporter activation at very high concentrations (≥ 100 mM) and therefore represent activation of the HSR via hyperosmotic stress, as previously described [46]. In general, the results for the HSE reporter cell line are in good agreement with data for HSPA1A reporter, or HSPA1A mRNA analysis in the literature [23,40].

**Fig 6:**
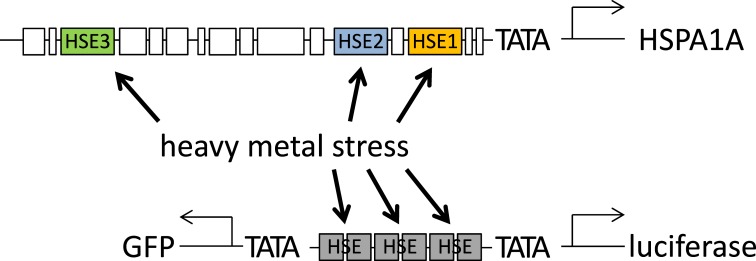
Activation of HSEs by heavy metals stress. Heavy metal stress acts on the heat shock pathway by activating HSF1 which interacts with the HSEs of target genes. Of key importance in this respect are affinity and position of the HSEs within the target promoter. Consensus HSEs were used to generate a high sensitivity reporter construct for detection of heavy metals.

In summary, the HSE reporter cell line C5 is a useful tool to detect and quantify HSF1 mediated HSR under different stress conditions (Fig 6). The use of HSP70 as a marker for heavy metal induced cell stress has already been suggested previously [25,26,37,38]. Therefore, an implementation of our optimized HSR reporter cell line into toxicological and eco-toxicological screenings could improve test strategies due to its sensitivity and possibility to easily and quantitatively compare HSR activation between samples.

## Supporting information

S1 Table Description of plasmids. (PDF)

S1 Fig Different heavy metals induce HSP72. HeLa cells were treated with either 50 μM CdSO4, 4 mM CuSO4 or 35 μM HgCl2 in DMEM with 10% FCS for 24 hours or left untreated. Whole cell extracts were prepared, and 5 μg of total protein were loaded per lane and separated on a 12% PAGE followed by western blot analysis. HSP72 induction was visualized by antibodies specific to HSP70 (Santa Cruz sc-1060-R, 1:10000) and GAPDH (Santa Cruz sc-25778, 1:5000) was used as a loading control.
(PDF)

S2 Fig HSE Reporter activity depends on the presence of HSF1. The knock-down of HSF1 with shRNA was demonstrated with Western Blot (A). Asterisk indicates an unspecific band. For analysis with CdSO_4_ (B) HEK 293 wildtype cells and HSF1 KD (XshHSF1-5-13) cells were transiently transfected with luciferase reporter (pMlucM 6HSE) and 24 h later treated with 50 μM CdSO_4_ in DMEM complete for 6 h. Y-axis shows relative luciferase activity compared to untreated control cells. All values show means of at least three independent experiments, with 12 technical replicates per plate. Error bars indicate SEM.
(PDF)

S3 Fig Induction of HSPA1A promoter containing mutated HSEs by heavy metals. Effect of individual HSEs on HSPA1A promoter activity analysed by transient transfection experiments in HEK 293 cells. Different promoter variations are all in the same plasmid background (pM Nluc PAUM; see S1 Table), where an (m) following the number of the HSE in the name indicates a mutation. HSR was induced by heat treatment at 42°C for 10 minutes (A) or by incubation with 50 μM CdSO_4_ (B), 75 μM HgCl_2_ (C) and 4 mM CuSO_4_ (D) for 1 h. All cells were recovered for 6 h after treatment. Cells were lysed, and luciferase activity was measured. Values shown are means of at least 3 independent experiments with 6–12 technical replicates each. Y-axis shows relative luciferase activity compared to untreated control cells. Error bars indicate SEM.
(PDF)

S4 Fig Mutations of HSEs in the HSPA1A promoter induced by HS. (A-C) Effects of individual HSEs on HSPA1A promoter activity analysed by transient transfection experiments in HEK 293 cells; induction with HS at 42°C for 10 min, followed by 6 h recovery at 37°C. All constructs in the same plasmid background (pM Nluc PAUM; see S1 Table). (A) Isolated HSEs, 3-fold multimerised; (B) combinations of the HSEs with or without spacers, compared to wildtype HSPA1A and (C) mutations of HSEs in the context of the promoter, (m) following the number of the HSE in the name indicates a mutation. P-values in (B) were calculated relative to HSE321. Values shown are means of 6 to 12 technical replicates and at least 3 independent experiments. Y-axis shows relative luciferase activity compared to untreated control cells. Error bars indicate SEM.
(PDF)

S5 Fig Analysis of C5 cells with Pb. C5 cells were treated with Pb(NO3)2 for 1 h. Then the cells were recovered for 6 h before luciferase measurement. Y-axis shows relative luciferase activity compared to untreated control cells.
(PDF)

S6 Fig HSPA1 induction after heavy metal treatment. C5 cells were treated with CuSO_4_, HgCl_2_, ZnCl_2_ or NiCl_2_ for 1 h and afterwards recovered for 2 h before mRNA harvest. Quantitative PCR was performed for HSPA1A/1B. GAPDH was used for normalization. Y-axis shows x-fold mRNA levels compared to untreated control cells. All values show means of at least three independent experiments.
(PDF)
