# Supplementary material for: HSF1 mediated stress response of heavy metals
Source: PLoS One. 2018 Dec 19;13(12):e0209077. doi: 10.1371/journal.pone.0209077 (PMC6300263; doi:10.1371/journal.pone.0209077)
Supplement: S1 Table — (PDF) [file pone.0209077.s001.pdf]

**plasmid**	**promoter elements**	**reporter**
**pM luc M 6HSE**	6 x HSE consensus	Fluc
**pMNluc PAUM 3xHSE**	3 x HSE consensus	NlucPAU
**pMNluc PAUM 3xHSE1**	3 x HSE1	NlucPAU
**pMNluc PAUM 3xHSE2**	3 x HSE2	NlucPAU
**pMNluc PAUM 3xHSE3**	3 x HSE3	NlucPAU
**pMNluc PAUM HSPA1A**	HSPA1A promoter (−712 to +1)	NlucPAU
**pMNluc PAUM HSE321**	1 x HSE3, HSE2 and HSE1 (short spacer)	NlucPAU
**pMNluc PAUM HSE321space**	1 x HSE3, HSE2 and HSE1 including long spacer sequences corresponding to their positions in the HSPA1A promoter	NlucPAU
**pMNluc PAUM 1m2m3m**	HSPA1A promoter with point mutations in HSE1, HSE2 and HSE3	NlucPAU
**pMNluc PAUM 12m3m**	HSPA1A promoter with point mutations in HSE2 and HSE3	NlucPAU
**pMNluc PAUM 1m23m**	HSPA1A promoter with point mutations in HSE1 and HSE3	NlucPAU
**pMNluc PAUM 1m2m3**	HSPA1A promoter with point mutations in HSE1 and HSE2	NlucPAU
**pMNluc PAUM 123m**	HSPA1A promoter with point mutations in HSE3	NlucPAU

S1 Table. Description of plasmids.
