# Supplementary material for: HSF1 mediated stress response of heavy metals
Source: PLoS One. 2018 Dec 19;13(12):e0209077. doi: 10.1371/journal.pone.0209077 (PMC6300263; doi:10.1371/journal.pone.0209077)
Supplement: S1 Fig — HeLa cells were treated with either 50 μM CdSO4, 4 mM CuSO4 or 35 μM HgCl2 in DMEM with 10% FCS for 24 hours or left untreated. Whole cell extracts were prepared, and 5 μg of total protein were loaded per lane and separated on a 12% PAGE followed by western blot analysis. HSP72 induction was visualized by antibodies specific to HSP70 (Santa Cruz sc-1060-R, 1:10000) and GAPDH (Santa Cruz sc-25778, 1:5000) was used as a loading control. (PDF) [file pone.0209077.s002.pdf]

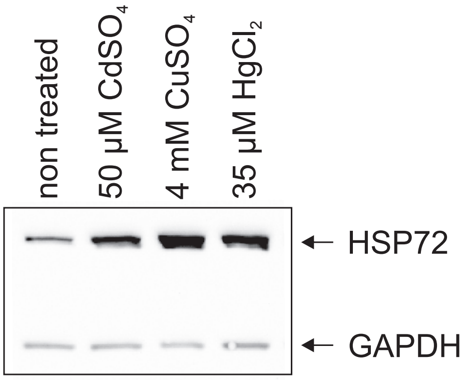

S1 Fig. Different heavy metals induce HSP72. HeLa cells were treated with either 50 µM $CdSO_4$, 4 mM $CuSO_4$ or 35 µM $HgCl_2$ in DMEM with 10% FCS for 24 hours or left untreated. Whole cell extracts were prepared, and 5 µg of total protein were loaded per lane and separated on a 12% PAGE followed by western blot analysis. HSP72 induction was visualized by antibodies specific to HSP70 (Santa Cruz sc-1060-R, 1:10000) and GAPDH (Santa Cruz sc-25778, 1:5000) was used as a loading control.
