# Supplementary material for: HSF1 mediated stress response of heavy metals
Source: PLoS One. 2018 Dec 19;13(12):e0209077. doi: 10.1371/journal.pone.0209077 (PMC6300263; doi:10.1371/journal.pone.0209077)
Supplement: S2 Fig — The knock-down of HSF1 with shRNA was demonstrated with Western Blot (A). Asterisk indicates an unspecific band. For analysis with CdSO4 (B) HEK 293 wildtype cells and HSF1 KD (XshHSF1-5-13) cells were transiently transfected with luciferase reporter (pMlucM 6HSE) and 24 h later treated with 50 μM CdSO4 in DMEM complete for 6 h. Y-axis shows relative luciferase activity compared to untreated control cells. All values show means of at least three independent experiments, with 12 technical replicates per plate. Error bars indicate SEM. (PDF) [file pone.0209077.s003.pdf]

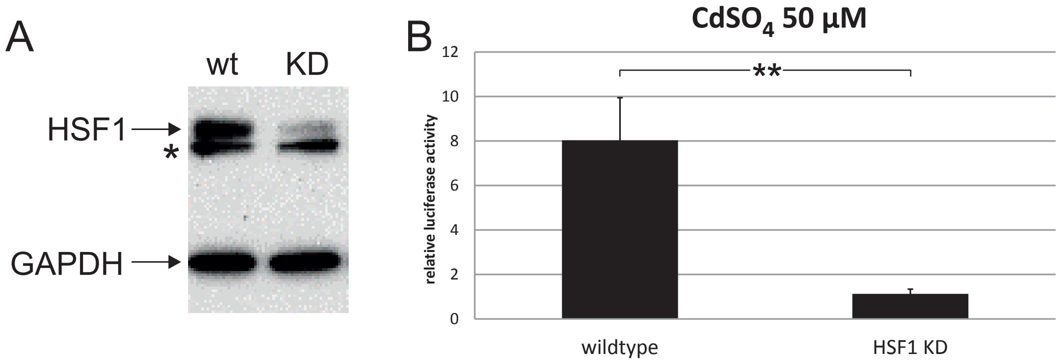


**S2 Fig. HSE Reporter activity depends on the presence of HSF1.** The knock-down of HSF1 with shRNA was demonstrated with Western Blot (A). Asterisk indicates an unspecific band. For analysis with $CdSO_4$ (B) HEK 293 wildtype cells and HSF1 KD (XshHSF1-5-13) cells were transiently transfected with luciferase reporter (pMlucM 6HSE) and 24 h later treated with 50 µM $CdSO_4$ in DMEM complete for 6 h. Y-axis shows relative luciferase activity compared to untreated control cells. All values show means of at least three independent experiments, with 12 technical replicates per plate. Error bars indicate SEM.
