# Supplementary material for: HSF1 mediated stress response of heavy metals
Source: PLoS One. 2018 Dec 19;13(12):e0209077. doi: 10.1371/journal.pone.0209077 (PMC6300263; doi:10.1371/journal.pone.0209077)
Supplement: S3 Fig — Effect of individual HSEs on HSPA1A promoter activity analysed by transient transfection experiments in HEK 293 cells. Different promoter variations are all in the same plasmid background (pM Nluc PAUM; see S1 Table), where an (m) following the number of the HSE in the name indicates a mutation. HSR was induced by heat treatment at 42°C for 10 minutes (A) or by incubation with 50 μM CdSO4 (B), 75 μM HgCl2 (C) and 4 mM CuSO4 (D) for 1 h. All cells were recovered for 6 h after treatment. Cells were lysed, and luciferase activity was measured. Values shown are means of at least 3 independent experiments with 6–12 technical replicates each. Y-axis shows relative luciferase activity compared to untreated control cells. Error bars indicate SEM. (PDF) [file pone.0209077.s004.pdf]

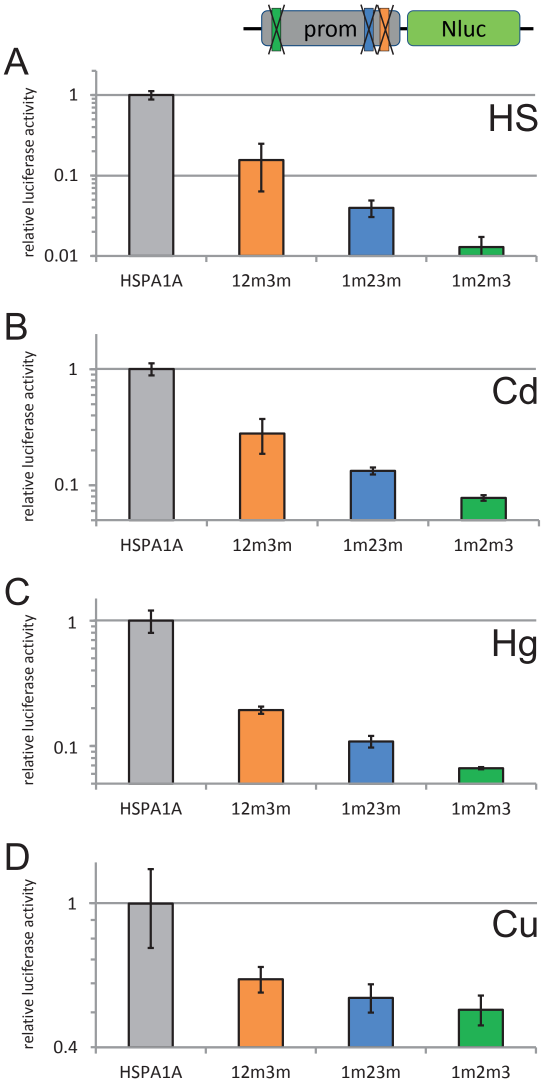

S3 Fig. Induction of HSPA1A promoter containing mutated HSEs by heavy metals. Effect of individual HSEs on HSPA1A promoter activity analysed by transient transfection experiments in HEK 293 cells. Different promoter variations are all in the same plasmid background (pM Nluc PAUM; see Table S1), where an (m) following the number of the HSE in the name indicates a mutation. HSR was induced by heat treatment at 42°C for 10 minutes (A) or by incubation with 50 µM $CdSO_4$ (B), 75 µM $HgCl_2$ (C) and 4 mM $CuSO_4$ (D) for 1 h. All cells were recovered for 6 h after treatment. Cells were lysed, and luciferase activity was measured.Values shown are means of at least 3 independent experiments with 6-12 technical replicates each. Y-axis shows relative luciferase activity compared to untreated control cells. Error bars indicate SEM.
