# Supplementary material for: HSF1 mediated stress response of heavy metals
Source: PLoS One. 2018 Dec 19;13(12):e0209077. doi: 10.1371/journal.pone.0209077 (PMC6300263; doi:10.1371/journal.pone.0209077)
Supplement: S4 Fig — (A-C) Effects of individual HSEs on HSPA1A promoter activity analysed by transient transfection experiments in HEK 293 cells; induction with HS at 42°C for 10 min, followed by 6 h recovery at 37°C. All constructs in the same plasmid background (pM Nluc PAUM; see S1 Table). (A) Isolated HSEs, 3-fold multimerised; (B) combinations of the HSEs with or without spacers, compared to wildtype HSPA1A and (C) mutations of HSEs in the context of the promoter, (m) following the number of the HSE in the name indicates a mutation. P-values in (B) were calculated relative to HSE321. Values shown are means of 6 to 12 technical replicates and at least 3 independent experiments. Y-axis shows relative luciferase activity compared to untreated control cells. Error bars indicate SEM. (PDF) [file pone.0209077.s005.pdf]

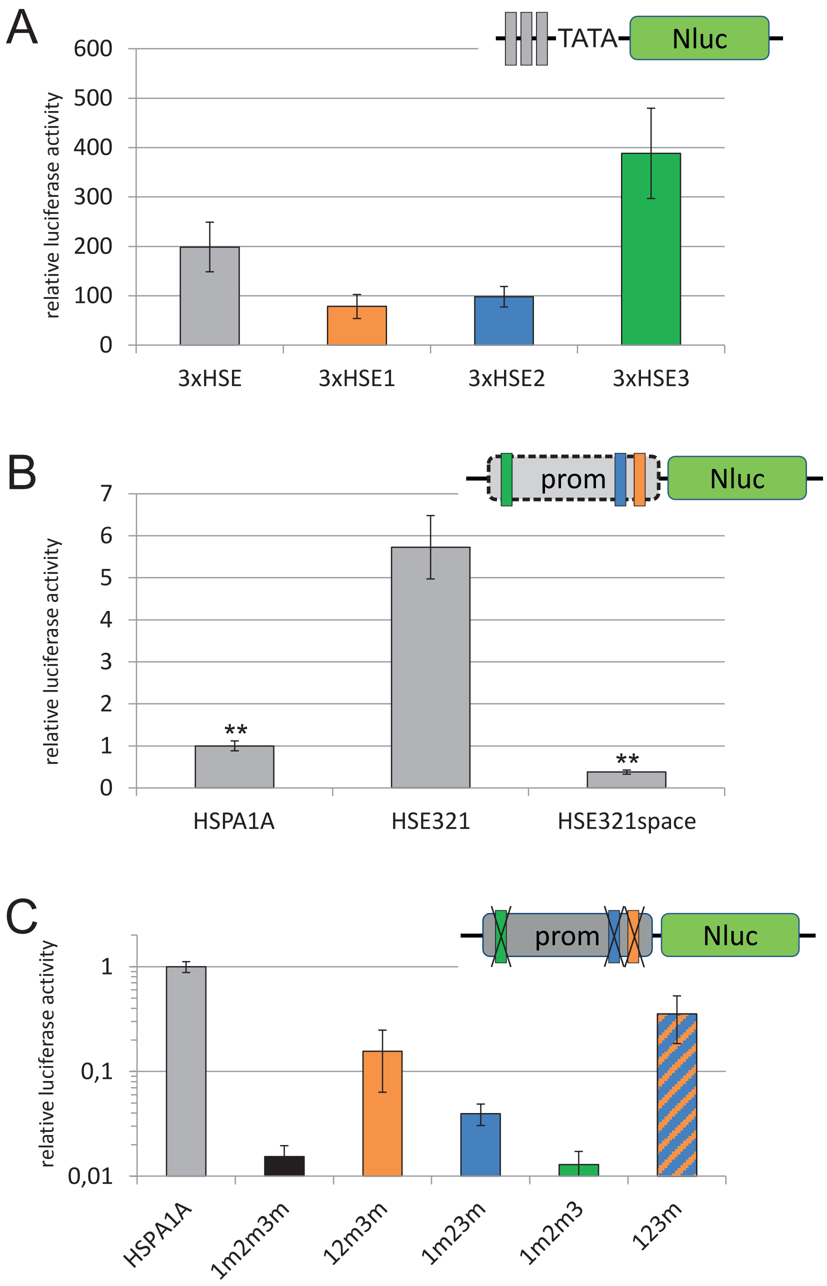

S4 Fig. Mutations of HSEs in the HSPA1A promoter induced by HS. (A-C) Effects of individual HSEs on HSPA1A promoter activity analysed by transient transfection experiments in HEK 293 cells; induction with HS at 42°C for 10 min, followed by 6 h recovery at 37 °C. All constructs in the same plasmid background (pM Nluc PAUM; see Table S1). (A) Isolated HSEs, 3-fold multimerised; (B) combinations of the HSEs with or without spacers, compared to wildtype HSPA1A and (C) mutations of HSEs in the context of the promoter, (m) following the number of the HSE in the name indicates a mutation. P-values in (B) were calculated relative to HSE321. Values shown are means of 6 to 12 technical replicates and at least 3 independent experiments. Y-axis shows relative luciferase activity compared to untreated control cells. Error bars indicate SEM.
