# Supplementary material for: HSF1 mediated stress response of heavy metals
Source: PLoS One. 2018 Dec 19;13(12):e0209077. doi: 10.1371/journal.pone.0209077 (PMC6300263; doi:10.1371/journal.pone.0209077)
Supplement: S5 Fig — C5 cells were treated with Pb(NO3)2 for 1 h. Then the cells were recovered for 6 h before luciferase measurement. Y-axis shows relative luciferase activity compared to untreated control cells. (PDF) [file pone.0209077.s006.pdf]

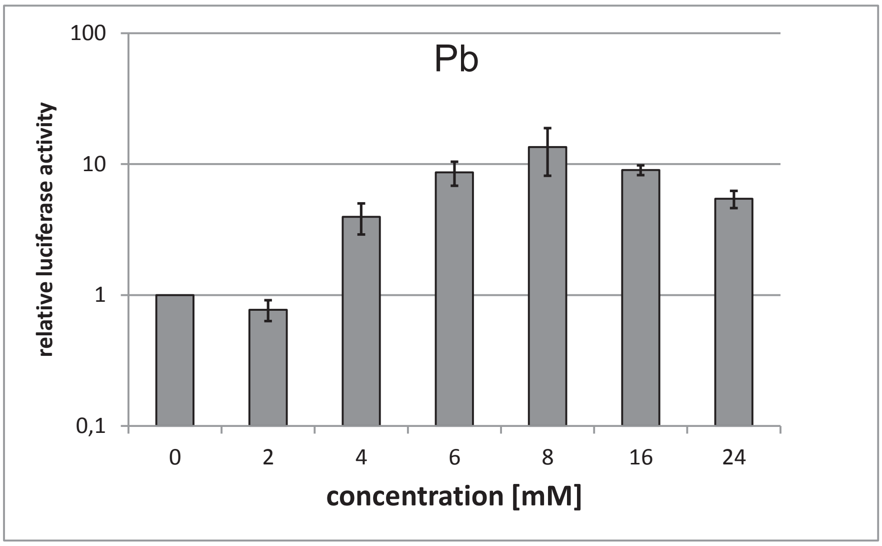

S5 Fig. Analysis of C5 cells with Pb. C5 cells were treated with $Pb(NO_3)_2$ for 1 h. Then the cells were recovered for 6 h before luciferase measurement. Y-axis shows relative luciferase activity compared to untreated control cells.
