# Supplementary material for: HSF1 mediated stress response of heavy metals
Source: PLoS One. 2018 Dec 19;13(12):e0209077. doi: 10.1371/journal.pone.0209077 (PMC6300263; doi:10.1371/journal.pone.0209077)
Supplement: S6 Fig — C5 cells were treated with CuSO4, HgCl2, ZnCl2 or NiCl2 for 1 h and afterwards recovered for 2 h before mRNA harvest. Quantitative PCR was performed for HSPA1A/1B. GAPDH was used for normalization. Y-axis shows x-fold mRNA levels compared to untreated control cells. All values show means of at least three independent experiments. (PDF) [file pone.0209077.s007.pdf]

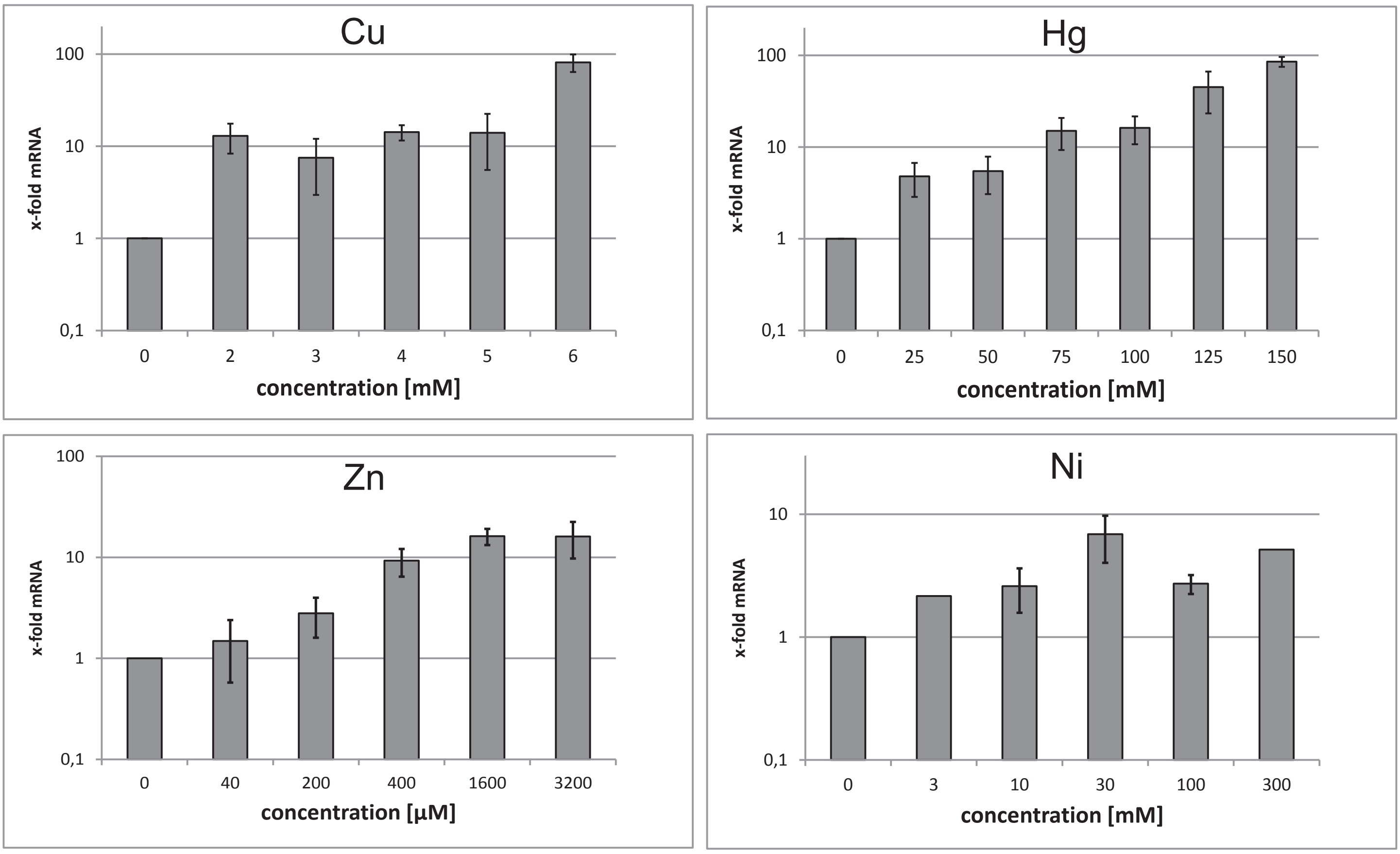


**S6 Fig**. **HSPA1 induction after heavy metal treatment.** C5 cells were treated with $CuSO_4$, $HgCl_2$, $ZnCl_2$ or $NiCl_2$ for 1 h and afterwards recovered for 2 h before mRNA harvest. Quantitative PCR was performed for HSPA1A/1B. GAPDH was used for normalization. Y-axis shows x-fold mRNA levels compared to untreated control cells. All values show means of at least three independent experiments. Error bars indicate SEM.
